# Comprehensive Analysis of Differentially Expressed mRNAs, lncRNAs and circRNAs Related to Intramuscular Fat Deposition in Laiwu Pigs

**DOI:** 10.3390/genes13081349

**Published:** 2022-07-27

**Authors:** Jingxuan Li, Xueyan Zhao, Yanping Wang, Jiying Wang

**Affiliations:** Shandong Provincial Key Laboratory of Animal Disease Control and Breeding, Institute of Animal Science and Veterinary Medicine, Shandong Academy of Agricultural Sciences, Jinan 250108, China; 15927409635@163.com (J.L.); zhaoxueyan0102@163.com (X.Z.); wangyanping03@163.com (Y.W.)

**Keywords:** message RNA (mRNAs), long noncoding RNAs (lncRNAs), circular RNAs (circRNAs), intramuscular fat (IMF), pigs

## Abstract

Long noncoding RNAs (lncRNAs) and circular RNAs (circRNAs) are important classes of small noncoding RNAs that can regulate numerous biological processes. To understand the role of message RNA (mRNAs, lncRNAs and circRNAs) in the regulation of intramuscular fat (IMF) deposition, in this study the expression profiles of longissimus dorsi (LD) muscle from six Laiwu pigs (three with extremely high and three with extremely low IMF content) were sequenced based on rRNA-depleted library construction. In total, 323 differentially expressed protein-coding genes (DEGs), 180 lncRNAs (DELs) and 105 circRNAs (DECs) were detected between the high IMF and low IMF groups. Functional analysis indicated that most DEGs, and some target genes of DELs, were enriched into GO terms and pathways related to adipogenesis, suggesting their important roles in regulating IMF deposition. In addition, 12 DELs were observed to exhibit a positive relationship with stearoyl-CoA desaturase (*SCD*), phosphoenolpyruvate carboxykinase 1 (*PCK1*), and adiponectin (*ADIPOQ*), suggesting they are highly likely to be the target genes of DELs. Finally, we constructed a source gene-circRNA-miRNA connective network, and some of miRNA of the network have been reported to affect lipid metabolism or adipogenesis. Overall, this work provides a valuable resource for further research and helps to understand the potential functions of lncRNAs and circRNAs in IMF deposition.

## 1. Introduction

Porcine intramuscular fat (IMF) is an essential determinant of meat quality in pig production, affecting the tenderness, tightness, flavor, and juiciness of pork. But in recent years, scholars have focused on improving pig growth rate and lean pork rate to achieve maximum economic value. This one-sided pursuit for high lean pork rate and growth rate has led to a decline in pork quality. On the other hand, with the development of the economy, people’s demand for meat tends to be more diversified. As one of the most consumed meat products, the quality of pork has attracted more and more attention. Therefore, appropriately increasing the IMF content has become more and more important. However, it is a challenge to directly select pigs with high IMF content due to the difficulty of in vivo measurement. Identifying candidate genes underlying and clarifying the molecular basis of IMF deposition are the key elements for improving pork quality.

Up to now, many research methods, such as candidate gene analyses [1], genome-wide microsatellite scanning [2], mRNA transcriptomics [3], and genome-wide association studies (GWAS) have been used to identify genes or regulators affecting porcine IMF deposition. However, few causative genes or reliable candidate genes were found for it. Further studies using new methods would be required to reveal the regulation mechanism of IMF deposition. In recent years, advances in sequencing technology have revealed that long noncoding RNAs (lncRNAs) and circular RNAs (circRNAs) have emerged as important class of gene expression regulators that can regulate numerous biological processes, such as cancer progression, neurological disorders, cancer cell proliferation, and muscle contraction [4,5,6,7,8,9,10]. Studies had proven that several lncRNAs and circRNAs are involved in adipogenesis. For instance, IncIMF2 acts as a molecular sponge for microRNA-217, which has been found associated with adipogenesis, thereby affecting the expression of the miR-217 target gene [11]. LncRNA IRLnc influence IMF decomposition by regulating the expression of nuclear receptor subfamily 4 group A member 3 [12]. In addition, lnc-ORA regulates adipogenesis through the phosphatidylinositol-3-kinase/protein kinase B/mechanistic target of rapamycin (PI3K/AKT/mTOR) signaling pathway in mice [13]. Additionally, accumulating evidence has indicated that circRNAs also play significant role in adipogenesis. For example, circSAMD4A regulated preadipocyte differentiation by acting as a miR-138-5p sponge, and thus increasing enhancer of zeste 2 polycomb repressive complex 2 subunit expression [14]. CircINSR could target miR-15/16 to inhibit the pre-mature differentiation of intramuscular preadipocytes [15]. CircFLT1 and lncCCPG1 sponges miR-93 to regulate the proliferation and differentiation of adipocytes in bovine [16]. These discoveries demonstrate the importance of lncRNAs and circRNAs in adipogenesis. However, our knowledge of lncRNAs and circRNAs in relation to porcine IMF deposition remains limited.

The Laiwu pig, as a representative fatty breed in North China, is characterized by high quality meat with high water-holding capacity, bright color, tender texture, and especially high IMF content (10.32%) [17]. Simultaneously, we found that there was a great difference in IMF content in Laiwu pigs (3.39–18.85%), as revealed by Huang in a study containing 333 Laiwu pigs [18]. Therefore, LW pig is an ideal experimental material for exploring the molecular mechanism of IMF deposition.

In this study, we collected longissimus dorsi (LD) muscle samples from 42 Laiwu pigs, then measured their IMF content; a total of six LW pigs (three with extremely high and three with extremely low IMF content from Jinan LW pig breeding Co. Ltd., Jinan, China) were selected. Subsequently, we performed comprehensive analysis of differentially expressed protein-coding genes (DEGs), lncRNAs (DELs) and circRNAs (DECs) related to IMF deposition in Laiwu pigs based on RNA-seq data using rRNA-depleted library construction. In the meantime, potential DEGs and DELs network and mRNA-circRNA-miRNA may affect IMF deposition were constructed.

## 2. Materials and Methods

### 2.1. Animals and Samples Preparation

Forty-two Laiwu pigs, including 29 castrated male and 13 female pigs, from Jinan Laiwu pig breeding Co. Ltd., Jinan, China, were slaughtered according to the standard process. LD muscle was collected at the thoracolumbar junction, and frozen in liquid nitrogen for RNA extraction. Meanwhile, about 200 g of LD muscle was also obtained around the thoracolumbar junction for the determination of IMF content. After removing the adipose and connective tissues, these muscle samples were oven dried to constant weight for removing moisture. Then the samples were ground, and IMF content was measured in triplicate per sample using the Soxhlet petroleum–ether method and expressed as the weight percentage of wet muscle tissue.

Taking gender, carcass weight and IMF content into account, three Laiwu pigs with extremely high IMF content and three Laiwu pigs with extremely low IMF content were selected for subsequent sequencing.

### 2.2. RNA Preparation, Library Construction and Sequencing

Total RNA was isolated from the LD muscles of the selected six pigs using TRIzol reagent (Invitrogen, Life Technologies, Waltham, MA, USA). Purity and concentration of total RNA were assessed according to the following three aspects: (1) Agarose gel electrophoresis was used to analyze the RNA integrity and the presence of DNA contamination; (2) Nanodrop 2000 spectrophotometer (Thermo Scientific, Waltham, MA, USA) was used for preliminary quantification to detect RNA concentration and purity, which was controlled in the range of 1.9–2.1; (3) Agilent 2100 Bioanalyzer (Agilent, Santa Clara, CA, USA) was used to accurately detect RNA integrity (RIN ≥ 7 and 28S/18S ≥ 0.7).

In total, 3 μg total RNA for each sample was used to construct sequencing libraries. A chain-specific library was constructed by removing ribosomal RNA. First, ribosomal RNA was removed from total RNA, and then the RNase R enzyme was used to break the RNA into short fragments of 250–300 bp. The first strand of cDNA was synthesized using the fragments as a template and random oligonucleotides as primers. Then, the RNA strand was degraded by RNase H. The second strand of cDNA was synthesized using dNTPs (dUTP, dATP, dGTP and dCTP) in the DNA polymerase I system. After the purification, the double strand cDNA was repaired at the end, the tail was added and the sequencing connector was connected. AMPure XP beads were used to screen the cDNA (350–400 bp). Uracil-Specific Excision Reagent enzyme was used to degrade the second strand of cDNA containing U, and then PCR amplification was performed to obtain the library.

Then, quality control was conducted on the libraries. After qualified, Illumina PE150 (Pair end 150 bp) sequencing was performed according to the effective concentration of the libraries and data production requirements of pooling.

### 2.3. Sequencing Data Quality Control, Transcriptome Assembly and Quantification of Gene Expression Level

By removing adapter reads, reads containing over 0.2% of poly-N, and low-quality paired reads (>50% of bases whose Phred scores were <5%) to filter raw reads. Then clean reads for subsequent analysis were obtained by filtering the raw data, checking the Phred score (Q20 and Q30) and GC content. Then the clean data were compared and analyzed by Hisat2 (https://daehwankimlab.github.io/hisat2/, accessed on 12 January 2022) using default parameters [19]. StringTie [20] was used for transcriptome assembly and reconstruction. At the same time, we used edgeR [21] to conduct differential expression analysis, and genes were considered as differentially expressed coding genes (DEGs) according to criteria, the *p* value < 0.01 and |log_2_(fold change)| ≥ 1.

### 2.4. lncRNAs Identification and Their Target Genes’s Prediction

According to the characteristics of lncRNA, the following four steps were used to identify lncRNA from the assembled transcript: (1) the single exon transcripts with low confidence in the splicing results were filtered and the transcripts with exon ≥ 2 were selected; (2) transcripts with a length > 200 nt were selected; (3) transcripts that overlap with the exon region of the database annotation were screened by using Cuffcompare [22]; (4) Coding potential calculator (CPC) analysis, coding-non-coding index (CNCI) analysis and protein families (PFAM) protein domain analysis were performed to identify lncRNAs. EdgeR [21] was also used to detect differential expression lncRNA (DELs) with the same criteria as DEGs

Target gene prediction is an important part of lncRNA analysis. We used two methods to predict target genes for DELs: (1) co-location: location-dependent target gene analysis. Cis target genes were predicted according to the positional relationship between lncRNAs and mRNAs, and the screening range was within 100 K; and (2) co-expression: expression related target gene analysis. Target genes were predicted according to the expression correlation between lncRNAs and mRNAs. The condition of screening was that the absolute value of correlation coefficient was greater than 0.95.

### 2.5. Identification of circRNAs and Analysis of circRNA-miRNA Binding Sites

Findcirc [23] was used to predict the circRNA. Anchor sequences of 20 nt were extracted from the two segments of reads that were not aligned to the reference sequence, and each pair of anchor sequences were aligned to the reference sequence again (the starting and ending sites were named as A3 and A4 respectively). If the 5′ end of the anchor sequences are aligned to the reference sequence, as well as the 3′ end of the anchor sequences are aligned to the upstream of this site (the starting and ending sites are named as A1 and A2 respectively), and there are splicing sites existed in A2 and A3; this read is considered as a candidate circRNA [24]. EdgeR [21] was also used to detect differential expression circRNA (DECs) with the criteria of the *p* value < 0.05 and |log_2_(fold change)| ≥ 1.

CircRNA can inhibit the function of miRNA by binding with miRNA [25]. In this study, miRanda software was used to predict the miRNA binding sites of circRNA. A total of 457 miRNAs downloaded from miRBase (Release 22.1, https://www.mirbase.org/, accessed on 5 February 2022) were used to analyze.

### 2.6. Functional Enrichment Analysis of DEGs, Target Genes of DELs and Source Genes of DECs

In order to predict the function of DEGs, target genes of DELs and source genes of DECs, Gene Ontology (GO) and Kyoto Encyclopedia of Genes and Genomes (KEGG) enrichment analysis were conducted using g: profiler (https://biit.cs.ut.ee/gprofiler/gost, accessed on 13 February 2022) with the criteria of FDR adjusted *p* value < 0.05.

Meanwhile, we also performed protein-protein interaction (PPI) network analysis. For the differential genes, the interaction between these differential genes was analyzed in the STRING database (https://string-db.org/, accessed on 25 March 2022), and further visualized with Cytoscape (http://www.cytoscape.org/, accessed on 28 March 2022).

### 2.7. Verification of Sequencing Data Using Reverse Transcription Quantitative PCR (RT-qPCR)

To verify the sequencing results, three DEGs and two DELs were selected for further confirmation by RT-qPCR method. PCR primers specific to the selected genes and the reference gene (18S) were designed using Primer Premier 5.0 (Applied Biosystems). Total RNA was reverse-transcribed into cDNA using RevertAid First Strand cDNA Synthesis Kit (Vazyme, Nanjing, China, R211-01). RT-qPCR for mRNA detection was carried out in Roche LightCyler 480 system (Roche, Mannheinm, Germany) with SYBR Green (Vazyme, Nanjing, China, Q711) according to the manufacturer’s instructions, The RT-qPCR data were analyzed using the 2^−ΔΔCT^ method, as previously described [26]. In brief, the relative fold changes of mRNA expression were quantified by normalizing the cycle threshold (CT) value of the experimental gene to the mean CT value of the reference gene 18S.

## 3. Results

### 3.1. IMF Content Detecting and Screening of Samples

In this study, LD muscle of 42 Laiwu pigs from a breeding farm of Shandong province, China, were collected and their IMF content were measured using Soxhlet extractor method. IMF content (Table S1 of Supplementary File S1) varied significantly among these individuals, ranging from 3.00% to 18.85% with an average of 9.00%.

Three Laiwu pigs with extremely high IMF content (S-H) and three Laiwu pigs with extremely low IMF (S-L) content were, respectively, selected for subsequent sequencing and analysis. The detailed information of the six individuals is provided in Table 1. All of the six individuals were the same gender (castrated male pigs). Compared with the other two groups (S-H vs. S-L), the average IMF content group of S-H group (16.66%) was significantly greater than that of S-L (4.59%) with a *p* value of 0.0032 (Table 1), whereas no significant difference existed in carcass weight (*p* value > 0.05).

### 3.2. Overview of Sequencing Data

In order to find the network pathways regulated by lncRNAs and systematically identify transcriptome differences between high and low IMF content Laiwu pigs, we constructed six cDNA libraries and sequenced them on the Illumina PE150 platform. Raw reads reached more than 78.6 million for each sequencing library. After removing adaptor reads, the proportion of N greater than 0.2% and low-quality contaminant reads; clean reads still exceeded 77.3 million. Then, the clean data were mapped to the Sus scrofa reference genome 11.1, and the ratio of mapped reads for each library was higher than 92% (Table 2). This result indicates the high-quality construction of cDNA libraries. Uniquely mapped reads were persevered to estimate the expression levels of genes.

### 3.3. Predictions and Analysis of mRNAs, lncRNAs and circRNAs

Stringtie was used to calculate the expression levels of all genes, which were described by fragments per kilobase per million reads (FKPM). A total of 21,363 known protein coding genes were detected by expression levels in the 6 cDNA libraries (Table S2 of Supplementary File S1).

**Figure 1:**
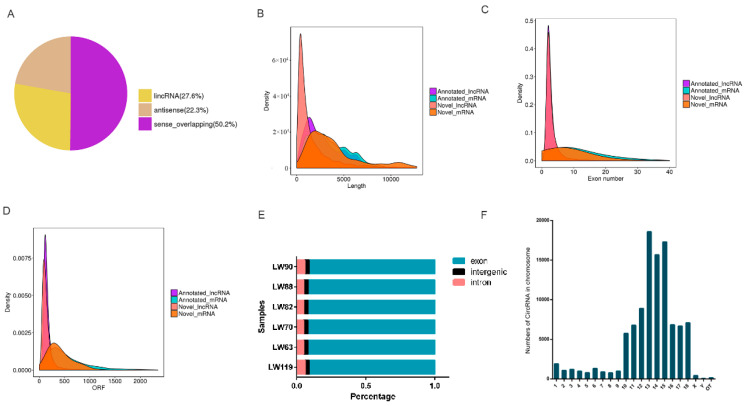
Characteristics of lncRNAs and circRNAs. (**A**) Summary of lncRNA types; (**B**) comparison of transcript size distribution; (**C**) comparison of exon number; (**D**) comparison of ORF length; (**E**) summary of circRNAs types; (**F**) the distribution of circRNA on the genomic location.

A total of 57,237 transcripts were detected, and 7386 lncRNAs were screened through three coding potential analysis methods (CPC2/PFAM/CNCI) (Table S3 of Supplementary File S1). The newly screened lncRNAs were divided into four types according to the position relationship with known mRNAs. The majority (50.2%) of the lncRNAs were sense-overlapping lncRNAs, while the minority (22.3%) were antisense lncRNAs (Figure 1A). The transcript length, exon number and open reading frame (ORF) length of lncRNAs and mRNAs were compared. As presented in Figure 1B–D, the transcript length, exon number, and ORF length of lncRNAs were less than those of mRNAs.

A total of 17,156 circRNAs were identified in the LD muscles of Laiwu pigs by Findcirc (Table S4 of Supplementary File S1). The distribution of circRNAs in different porcine genomes was roughly the same, with the average percentage of exonic circRNAs was 91.64%, while those of intergenic and intronic region circRNAs were 3.13% and 5.21% (Figure 1E). The circRNA genomic location on the chromosomes is shown in Figure 1F. It can be seen that these circRNAs are not uniformly distributed among chromosomes. Chr13 possessed the greatest number of circRNAs, followed by Chr15 and Chr14.

### 3.4. Differential Expression of mRNAs, lncRNAs and circRNAs

**Figure 2:**
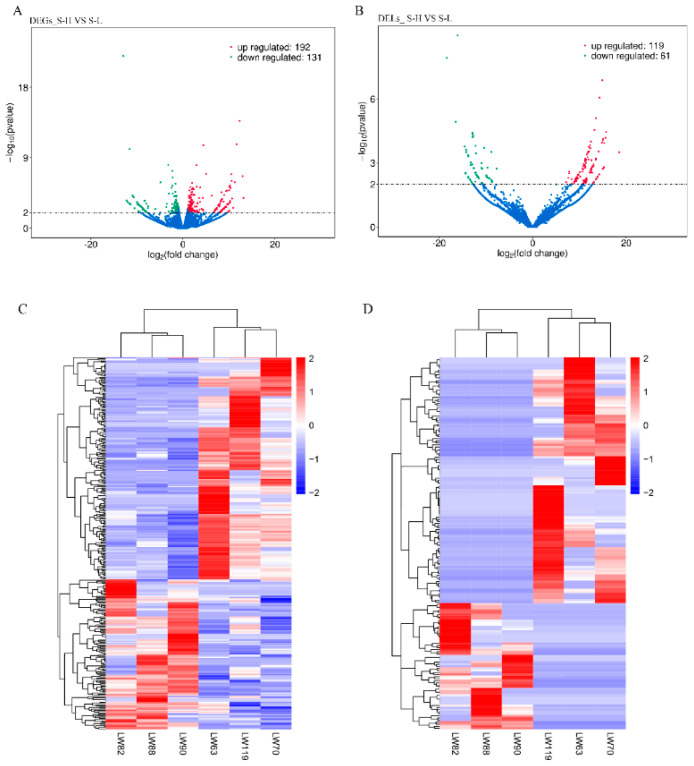
Comparative analysis of DEGs and DELs. (**A**) Volcano plot demonstrating a distinguishable DEGs expression pattern between S-H vs. S-L group. (**B**) Volcano plot demonstrating a distinguishable DELs expression pattern between S-H vs. S-L group. (**C**) Hierarchical clustering dendrogram analysis were conducted with DEGs among six different comparative groups. (**D**) Hierarchical clustering dendrogram analysis were conducted with DELs.

To explore the comprehensive transcriptional differences between high and low IMF groups (S-H vs. S-L), differentially expressed mRNAs, lncRNAs and circRNAs were detected, yielding 323 DEGs, 180 DELs and 105 DECs, respectively (Tables S5–S7 of Supplementary File S1, Figure 2A,B and Figure S1A).

To gain insights into the expression patterns of DEGs, DELs, and DECs in the libraries, we performed hierarchical cluster analysis based on expression abundance. As shown in the Figure 2C, LW63, LW70, LW119 clustered into S-H group, while LW82, LW88, LW90 divided into S-L group, indicating the expression patterns are consistent within high or low IMF content groups and different between the high and low groups. Likewise, DELs and DECs also showed distinguishable expression patterns between high and low IMF content groups (Figure 2D and Figure S1B).

### 3.5. Function Enrichment Analysis of DEGs, DELs and DECs

**Figure 3:**
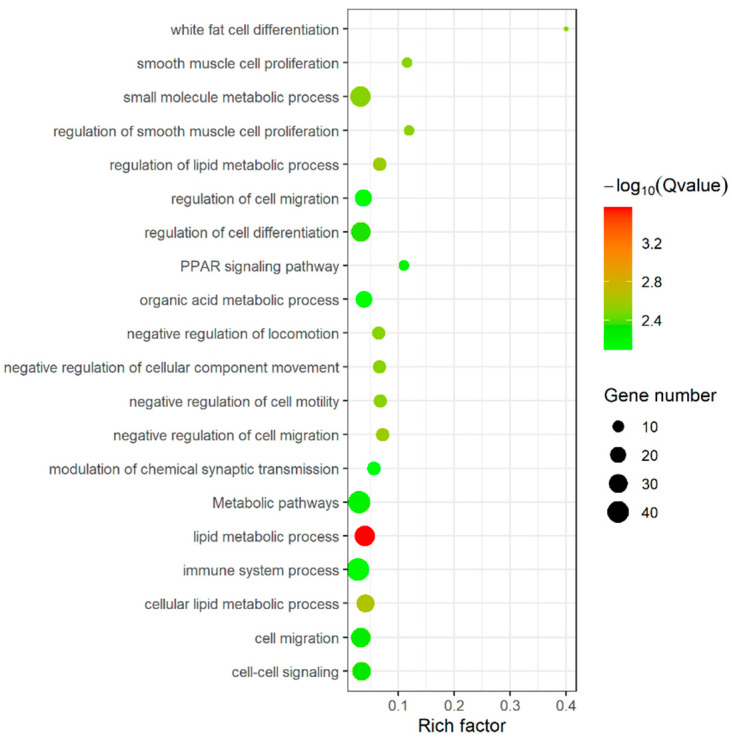
Functional analysis of DEGs: the most enriched top 20 Go terms and KEGG pathways of DEGs.

To further elucidate the functional roles of DEGs detected between high and low IMF group, we performed GO and KEGG pathway enrichment analyses of them. A total of 167 significant enriched GO terms were observed after FDR adjustment (adjusted *p* value < 0.05) for these DEGs detected (Table S8 of Supplementary File S1). Among the 167 GO terms, 161 were belonged to biological process (BP), and most of these BP terms were involved in lipid metabolic (lipid metabolic process, regulation of lipid metabolic process, and regulation of lipid transport), regulation of cell differentiation (white fat cell differentiation, brown fat cell differentiation, and regulation of smooth muscle cell proliferation), and biosynthesis (hexose biosynthetic process, monosaccharide biosynthetic process, and gluconeogenesis). Simultaneously, these DEGs were enriched into three KEGG pathways (adjusted *p* value < 0.05), including peroxisome proliferator-activated receptors (PPAR) signaling pathway, metabolic pathways, and adenosine 5′-monophosphate (AMP)-activated protein kinase (AMPK) signaling pathway (Figure 3), which were all key pathways related to adipogenesis. The results of function analysis indicate that there are a considerable number of DEGs which may play a certain role in adipogenesis.

Additionally, we used these two methods to predict and analyze target genes of DELs. In total, we obtained 1243 co-expression target genes and 733 co-location target genes of DELs, respectively (Table S9 of Supplementary File S1). Then, to have a functional view of DELs, we performed function enrichment analysis of these target genes using the same methods as DEGs. A total of 619 significant enriched GO terms, including 552 BP terms, 40 MF terms, and 27 cellular component (CC) terms, and 40 KEGG pathway were observed with the criterion of adjusted *p* value < 0.05 (Table S10 of Supplementary File S1). Most of the enriched terms and pathways were involved in immune response, and some were related with basic cell metabolism, such as cell activation, cell differentiation, intracellular signal transduction. Some terms and pathway associated with adipogenesis, such as lipid storage, PPAR signaling pathway, janus kinase-signal transducers and activators of transcription (Jak-STAT) signaling pathway, were also identified [27].

DECs’ function was also explored based on their source genes (Table S11 of Supplementary File S1). The results (Table S12 of Supplementary File S1) showed that no GO terms and pathways related to lipid metabolism were significantly enriched for DECs’ source genes.

### 3.6. Expression Regulation Analysis of DELs and Target Genes

**Figure 4:**
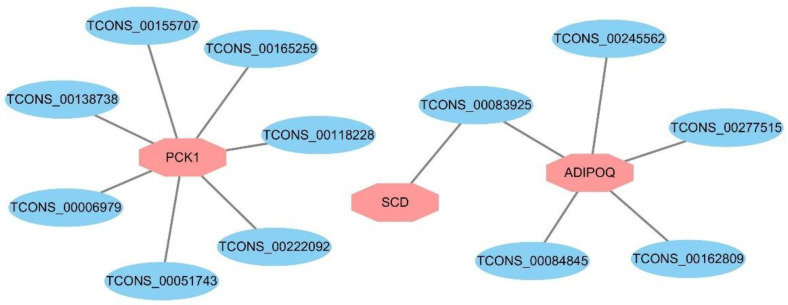
Correlation networks for DELs and their target genes. Rhombuses represent 3 target genes, ellipses represent 12 DELs.

To further investigate the relationship between DEGs and DELs, the target genes of DELs enriched in adipogenesis-related pathways were selected. Then, a review of the literature found that there are three genes, stearoyl-CoA desaturase (*SCD*), phosphoenolpyruvate carboxykinase 1 (*PCK1*) and adiponectin (*ADIPOQ*) affecting IMF deposition, and all of them came from the PPAR signaling pathway. Subsequently, DELs co-expressed and differentially expressed with these three genes were selected. Meanwhile, 12 DELs exhibited a positive relationship with the three genes (Figure 4). These three genes are highly likely to be the target genes of DELs, which requires further investigation.

### 3.7. CircRNA Functional Predictions

Previous studies have shown that circRNAs can act as miRNA sponges, thereby affecting the expression of miRNA target genes. Therefore, miRNA-circRNA binding sites analysis is helpful to further study the function of circRNAs.

**Figure 5:**
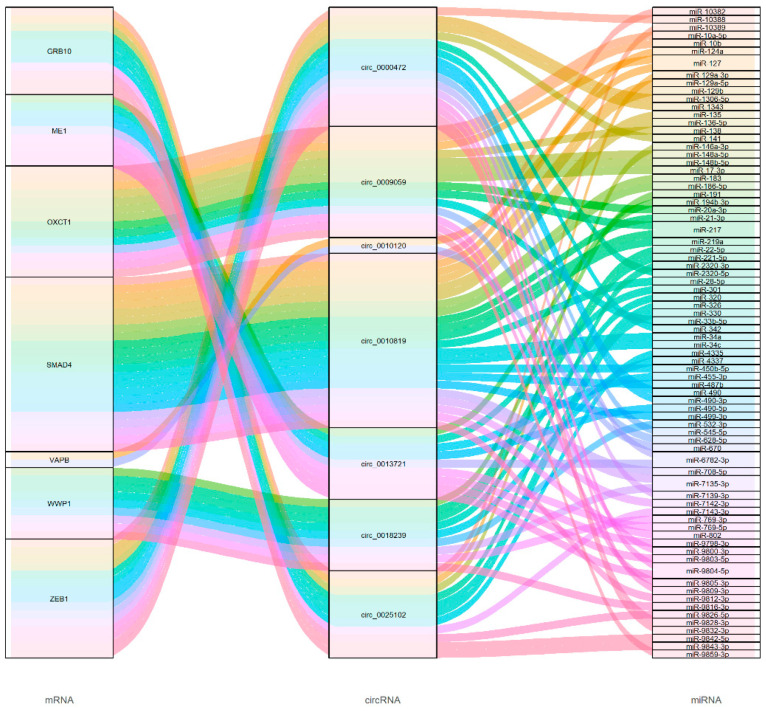
CircRNA network diagram for the source gene, DECs and their target miRNAs. Each rectangle represents a gene, and the connection degree of each gene is visualized based on the size of the rectangle.

DECs whose source genes were related adipogenesis were selected to carry out miRNA-circRNA binding sites analysis by miRanda software, including *circ_0025102*, *circ_0013721*, *circ_0009059* and so on. A basic source gene-circRNA-miRNA connective network was constructed (Figure 5), which can provide a reference for subsequent studies on the function of these DECs. The results showed that there were 82 potential interactions between above-mentioned DECs and 77 miRNAs.

### 3.8. Validation of DEGs and DELs

**Figure 6:**
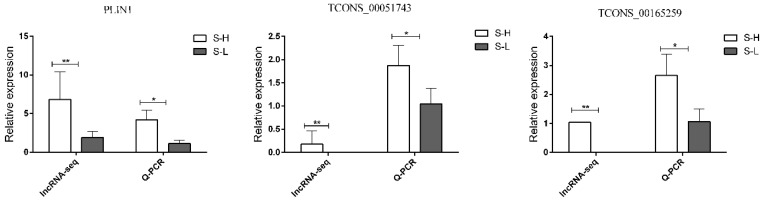
Verification of sequencing data by RT-qPCR. Comparisons of relative expression levels for 1 DEGs and 2 DELs. Comparisons of relative FPKM in the left; comparisons of relative expression levels in the right. * means *p* < 0.05, ** means *p* < 0.01. Since the FPKM values of *TCONS_00051743* and *TCONS_00165259* in the S-L group are 0, so there are no columns in the bar chart.

In order to technically validate the data generated by sequencing, we randomly selected several differentially expressed genes for quantitative verification, primers of three genes (Table S13 of Supplementary File S1) were designed, and validated by RT-qPCR using 18S as the reference gene. As demonstrated in Figure 6, RT-qPCR results of the two DELs (*TCONS-00165259* and *TCONS-00051743*) and one DEGs (*PLIN1*) were consistent with the sequencing data (Figure 6). This result demonstrated the high reliability and accuracy of RNA-seq gene expression data in this study.

## 4. Discussion

Pork is one of the most widely consumed meats in the world [28]. IMF content is an important indicator for pork quality evaluation, such as tenderness, flavor, and juiciness [29,30,31]. In animal production, we found that the range of measured IMF content of longissimus dorsi muscle derived from Laiwu pigs was large. Therefore, transcriptome analysis of extremely high and low IMF content comparison groups is suitable for identifying the novel candidate genes affecting IMF content.

In this study, we compared the expression profile of mRNAs, lncRNAs and circRNAs in LD muscles between Laiwu pigs with extremely high and low IMF content (S-H and S-L) through RNA-seq technology. A number of differentially protein-coding genes were detected to be differentially expressed in LD muscles with different IMF content, and identified DEGs are associated with normal physiological cell processes, including lipid metabolic process, cellular lipid metabolic process, negative regulation of cell migration, and adipogenesis pathways, including PPAR signaling pathway, AMPK signaling pathway and metabolic pathway. Many DEGs were reported to regulate adipogenesis or enriched in lipid metabolism-related pathways, such as *SCD*, fatty acid binding protein 4 (*FABP4*), *PLIN1*, Acetyl-CoA acyltransferase 1 (*ACAA1*), *ADIPOQ*, *PCK1*, and Peroxisome proliferator activated receptor γ (*PPARG*). For instance, *SCD* is a candidate key gene regulating IMF deposition, and has been reported to be critical for forming lipid droplets of 3T3-L1 adipocytes [28,32,33]. Lipid transporter *FABP4* acts as a fatty acids chaperone, which couples intracellular lipids to biological targets and signaling pathways, and extracellular *FABP4* can increase intracellular lipid accumulation [34,35].

Besides, *ACAA1* and *PLIN1* also play an important role in regulating IMF deposition. *ACAA1* is an important regulator of lipid metabolism and plays an essential role in fatty acid oxidation and lipid metabolism. Wang’s study findings revealed that *ACAA1* is closely associated with the PPAR signaling and fatty acid metabolism pathways, which are involved in fat deposition in sheep [36]. *PLIN1*, an adipose differentiation-related protein, is involved in the regulation of IMF content in pigs. It has been reported that the expression level of *PLIN1* was significantly higher in the high IMF content group when compared with the low IMF content group, and expression was increased during adipocyte differentiation [37]. In our study, *SCD*, *PLIN1*, and *FABP4* were all highly expressed in Laiwu pigs with high IMF content (S-H), which were highly consistent with previous reports.

In recent years, a large number of studies have shown that lncRNA plays a significant role in fat deposition. For instance, IMFNCR acts as a ceRNA to sequester miR-128-3p and miR-27b-3p, leading to heightened *PPARG* expression, and thus promotes intramuscular adipocyte differentiation [38]. Besides, lncRNA, Gm15290 sponges miR-27b to increase fat deposition and body weight in mice [39]. Therefore, lncRNA has attracted our attention. Studies have demonstrated that lncRNAs significantly influence gene regulation in cis [40,41,42]. lncRNA genes preferentially cooperate with protein-coding genes within the nearby ~100 kb region. Thus, the nearest neighboring protein-coding genes may contribute to interpreting lncRNA functions [43,44,45]. Besides, co-expression analysis was used to predict distant target genes. We explored the DELs function through GO and KEGG pathway analyses of their potential target genes, and observed that some target genes participated in some lipid metabolism-related BP terms or pathways, including positive regulation of fatty acid metabolic process, PPAR signaling pathway [46], Jak-STAT signaling pathway [27]. Thus, we speculate that some lncRNAs may participate in IMF deposition or adipogenesis by regulating their target genes.

In the meantime, we further performed expression regulation analysis between target genes and DELs enriched in IMF deposition-related pathways, and found that 12 DELs were strongly correlated with three DEGs related to IMF deposition, including *PCK1*, *SCD*, and *ADIPOQ* (Figure 4). It has been reported that *PCK1* promotes lipid accumulation in buffalo intramuscular adipocytes, which indicate the important role of *PCK1* in buffalo IMF deposition, and *PCK1* is a key gene of PPAR signaling pathway [47]. Some important candidate DELs, such as *TCON_00165259* and *TCON_00138738*, have high expression levels in our data and can target *PCK1*, could be considered as key candidate genes affecting IMF deposition in pigs. Besides, *SCD* and *ADIPOQ* are also pivotal genes that have been reported to affect adipogenesis [33,48]. The results suggest that these lncRNAs might influence IMF deposition by regulating their target genes.

Previous studies have shown that circRNAs are recognizable based on back-spliced reads in rRNA depleted RNA sequence data [49,50,51]. Meanwhile, a growing number of studies have found that circRNAs also play an important role in fat deposition. For example, circ-PLXAN1 can affect the differentiation of duck adipocytes and participate in fat deposition by binding miR-214 [52]. In addition, sus_circPAPPA2 regulates fat deposition in castrated pigs through the miR-2366/GK Pathway [53]. These studies illustrate the importance of circRNAs. Therefore, circRNAs in the sequencing data were also identified, and miRNA-circRNA binding sites analysis was conducted. Meanwhile, a basic source gene-circRNA-miRNA connective network (Figure 5) was constructed, which can provide a reference for subsequent studies on the function of these DECs. As shown in Figure 5, some of DECs’ target miRNA, such as miR-127, miR-217, and miR-183, have been reported to affect lipid metabolism or adipogenesis [11,54,55]. The results suggestthat circRNAs may be involved in the process of adipogenesis.

## 5. Conclusions

In summary, a genome-wide comparison of the expression profiles of mRNAs, lncRNAs, and circRNAs between S-H and S-L was investigated here. Functional analysis revealed that many mRNAs, lncRNAs, and circRNAs were involved in IMF deposition-related processes. Given that the role of lncRNAs and circRNAs in IMF deposition in pigs has not been fully elucidated, this work provides a valuable resource for further research and helps to understand the potential functions of lncRNAs and circRNAs.

## Figures and Tables

**Table 1:** Information of samples used in the study.

Group	Samples	Carcass Weight (kg)	Average (kg)	*p* Value of *t*-Test	IMF Content (%)	Average	*p* Value of *t*-Test
High IMF content group	LW63	77.7	74.6	0.1428	18.85	16.66	0.0032
	LW70	71.6			14.38		
	LW119	74.6			16.74		
Low IMF content group	LW82	66.4	70.0		5.90	4.59	
	LW88	72.5			3.39		
	LW90	71.0			4.49		

**Table 2:** Output statistics of sequencing reads.

Samples	Raw Reads	Clean Reads	Error Rate (%)	Total Mapped (%)	Uniquely Mapped (%)	Q20 (%)	Q30 (%)	GC Content (%)
LW119	99403460	97533328	0.02	95.11%	86.22%	98.11	94.66	51.26
W63	86027762	84512732	0.03	94.65%	83.13%	97.97	94.31	53.01
W70	83537954	82059898	0.02	94.67%	84.25%	98.05	94.51	53.27
LW82	89852982	87838194	0.03	94.26%	83.94%	97.88	94.11	51.55
LW88	78555518	77268288	0.02	92.08%	80.88%	97.96	94.39	53.68
LW90	99854922	98262148	0.02	94.72%	84.39%	98.07	94.58	52.47

## Data Availability

The raw data of RNA-seq of the LD muscles of the six Laiwu pigs have been deposited in the National Center for Biotechnology Information Sequence Read Archive with accession No. GSE207279 (Available online: https://www.ncbi.nlm.nih.gov/geo/query/acc.cgi?acc=GSE207279).

## Supplementary Materials

The following are available online at https://www.mdpi.com/article/10.3390/genes13081349/s1, Figure S1: Differential analysis and functional analysis of DECs. Supplementary File S1: Table S1: Information of samples used in the study. Table S2: Detailed information of protein coding genes. Table S3: Detailed information of lncRNA. Table S4: Detailed information of circRNA. Table S5: Detailed information of differentially expressed coding genes (DEGs) detected between high and low IMF content groups. Table S6: Detailed information of differential expression lncRNA (DELs) detected between high and low IMF content groups. Table S7: Detailed information of differential expression circRNA (DECs) detected between high and low IMF content groups. Table S8: Enriched GO terms and KEGG pathways of differential expression protein-coding genes (DEGs) detected between high and low IMF content groups. Table S9: Target genes of differential expression lncRNA (DELs) detected between high and low IMF content groups. Table S10: Target genes of differential expression lncRNA (DELs) detected between high and low IMF content groups. Table S11: Source genes of differential expression circRNA (DECs) detected between high and low IMF content groups. Table S12: Enriched GO terms and KEGG pathways of target genes of differential expression circRNA (DECs) detected between high and low IMF content groups. Table S13: Primers used for RT-qPCR verification.

## Abbreviations

IMF	intramuscular fat
lncRNAs	long noncoding RNAs
circRNAs	circular RNAs
LD	longissimus dorsi
DEGs	differentially expressed protein-coding genes
DELs	differentially expressed lncRNAs
DECs	differentially expressed circRNAs
GO	gene ontology
KEGG	kyoto encyclopedia of genes and genomes
CT	cycle threshold
*SCD*	stearoyl-CoA desaturase
*PCK1*	phosphoenolpyruvate carboxykinase 1
*ADIPOQ*	adiponectin
*FABP4*	fatty acid binding protein 4
*ACAA1*	acetyl-coa acyltransferase 1
*PLIN1*	perilipin 1
*PPARG*	peroxisome proliferator activated receptor γ
PPAR	peroxisome proliferator-activated receptors
AMPK	adenosine 5′-monophosphate (AMP)-activated protein kinase
Jak-STAT	janus kinase-signal transducers and activators of transcription
PI3K/AKT/mTOR	phosphatidylinositol-3-kinase/protein kinase B/mechanistic target of rapamycin
